# AΙ-Driven Drug Repurposing: Applications and Challenges

**DOI:** 10.3390/medicines12040028

**Published:** 2025-11-13

**Authors:** Paraskevi Keramida, Nikolaos K. Syrigos, Marousa Kouvela, Garyfallia Poulakou, Andriani Charpidou, Oraianthi Fiste

**Affiliations:** 1 Oncology Unit, Third Department of Internal Medicine and Laboratory, Medical School, National and Kapodistrian University of Athens, 11527 Athens, Greece; nksyrigos@gmail.com (N.K.S.); markouvela@yahoo.gr (M.K.); dcharpidou@yahoo.gr (A.C.); ofiste@med.uoa.gr (O.F.); 2 Third Department of Internal Medicine and Laboratory, Medical School, National and Kapodistrian University of Athens, 11527 Athens, Greece; gpoulakou@med.uoa.gr

**Keywords:** drug repurposing, artificial intelligence, machine learning, drug development

## Abstract

Drug repurposing is the process of discovering new therapeutic indications for already existing drugs. By using already approved molecules with known safety profiles, this approach reduces the time, costs, and failure rates associated with traditional drug development, accelerating the availability of new treatments to patients. Artificial Intelligence (AI) plays a crucial role in drug repurposing by exploiting various computational techniques to analyze and process big datasets of biological and medical information, predict similarities between biomolecules, and identify disease mechanisms. The purpose of this review is to explore the role of AI tools in drug repurposing and underline their applications across various medical domains, mainly in oncology, neurodegenerative disorders, and rare diseases. However, several challenges remain to be addressed. These include the need for a deeper understanding of molecular mechanisms, ethical concerns, regulatory requirements, and issues related to data quality and interpretability. Overall, AI-driven drug repurposing is an innovative and promising field that can transform medical research and drug development, covering unmet medical needs efficiently and cost-effectively.

## 1. Introduction

Drug repurposing, also known as drug repositioning, reprofiling, redirecting, or rediscovery, is the process of reusing already approved drugs or active substances for indications different from their original use [1,2]. Traditional drug development is a lengthy, costly, and multi-stage process, from target identification to clinical trials. The first step of target identification and validation is to recognize a suitable molecule or pathway that plays a key role in the disease pathophysiology, making it a possible therapeutic object. Cellular analysis by using genomics and proteomics methods along with biomedical informatics contributes to the completion of the first stage. Secondly, compound screening aims to distinguish the particles, which are more active towards the target, among huge libraries of compounds using experimental (combinatorial chemistry and high-throughput screening) and computational (virtual screening) techniques. The subsequent phase is preclinical studies in which both the pharmacokinetics and toxicity are tested in experimental animals. The last but not least step is the administration of the potential drug to a group of humans within the context of a clinical trial, consisting of three phases. Phase I assesses the drug safety in a few healthy volunteers. Phase II estimates the effectiveness and safety of the candidate drug in a number of patients, and Phase III continues the efficiency and toxicity testing but on a larger scale of individuals affected by the same ailment. After the successful completion of the above phases, competent authorities examine the marketing approval of the new drug and its public access [3]. According to a study published in the Journal of Health Economics, the total cost of developing a new drug is approximately $2.6 billion and it takes about 10 to 15 years to reach public access [4]. On the other hand, a repurposed drug seems to gain access to the market with approximately US $300 million [5]. Repurposed drugs benefit from existing preclinical and clinical data, requiring less time for approval (at least 3 years with the lowest average duration at 6 years) and carrying a lower risk of failure in clinical trials [6]. Proven safe drugs with pharmacodynamic and pharmacokinetic characteristics appropriate for human use that were not approved for a specific indication best fit for reprofiling [7]. The practice of repurposing is especially useful in diseases with restricted or nontherapeutic options, called rare or orphan diseases, as it diminishes the failure rate of drug production and speeds up the patient’s access to a promising treatment [8]. Another challenge that drug repositioning contributes to face is public health crisis, like the COVID-19 pandemic, where the use of repurposed drugs accelerated the therapeutic process [9]. Nowadays, Artificial Intelligence (AI) tools can manage and process big amounts of complicated datasets making the drug repurposing procedure quicker, more effective and facilitated [9,10]. Over time, many drugs have been successfully used as repurposed drugs like sildenafil (Viagra), which was originally examined for angina and finally gained approval for erectile dysfunction, minocycline, and dimethyl fumarate [11]. An example that arises from COVID-19 pandemic is baricitinib. This drug, which was originally approved to treat rheumatoid arthritis due to its anti-inflammatory properties, was repurposed for COVID-19 treatment following promising clinical trial outcomes [12].

## 2. Materials and Methods

This review was conducted by performing a detailed literature search in the bibliographic databases PubMed and Google scholar. The purpose was to indicate and analyze relevant studies on drug repurposing, published in international journals. The search was performed by using MeSH words, such as “drug-repurposing”, “artificial intelligence”, “AI”, and “machine learning”. Boolean operators, mainly “AND” and “OR”, used to redirect the search parameters and expand the conceptual coverage of the topic. The included studies were published in English and focused on the AI tools, their contribution in drug repurposing, and their applications in various medical fields. Studies published in the last decade were preferred. Studies published in languages other than English were excluded. Publications before 2015 were not a priority unless they provided substantial information about the topic. Moreover, information was drawn from books and reliable websites.

## 3. AI Tools in Drug Repurposing

John McCarthy first introduced the pair of words “Artificial Intelligence-AI” in 1956 [13]. This term refers to the capability of computer systems or algorithms to imitate intelligent human behavior [14]. So, modern AI tools are capable of handling large and complex datasets, facilitating the drug repurposing process and making it more efficient. By integrating diverse biomedical data, they can identify non-obvious drug–disease associations and accelerate the discovery of novel applications for the existing drugs, while simultaneously reducing time and costs compared with traditional drug development methods [9,10]. This highlights the transformative impact of AI-driven approaches on drug repurposing and their promise to improve patient access to effective treatments.

### 3.1. Machine Learning (ML)

**Figure 1:**
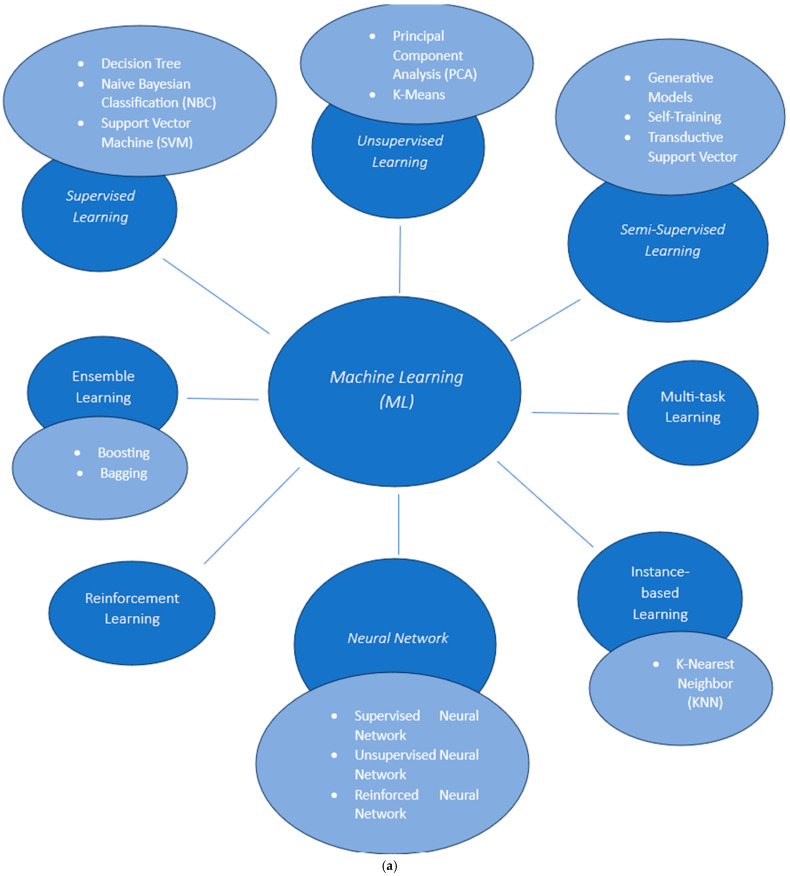

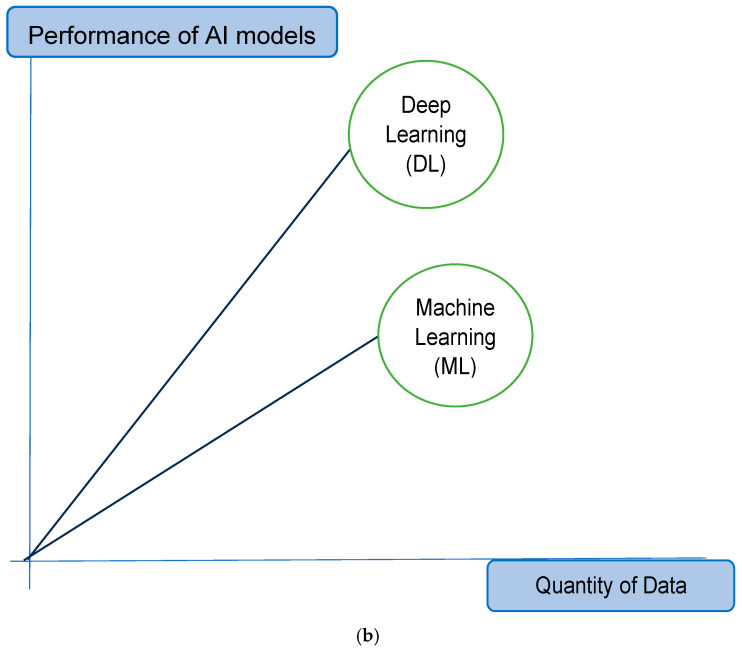
(**a**): Types of ML algorithms. (Based on “Machine Learning Algorithms—A Review”, Maseh, B. [15]). Note that the figure describes a simplified classification; some algorithms may be applicable across multiple categories depending on the conditions. (**b**): ML vs. DL performance correlated with the quantity of data (Based on “Machine Learning: Algorithms, Real-World Applications and Research Directions”, Sharker, I.H. [24]).

Machine learning (ML) is a set of algorithms which helps computers to learn from the inserted data, empirically, without programming being necessary. In this way, machines are educated to handle and analyze the big quantities of data by their own [15]. Representative ML algorithms are Logistic Regression (LR), Naive Bayesian Classification (NBC), k-Nearest Neighbor (KNN), Multiple Linear Regression (MLR), Support Vector Machine (SVM), Probabilistic Neural Network (PNN), Binary Kernel Discrimination (BKD), Linear Discriminant Analysis (LDA), Random Forest (RF), Artificial Neural Network (ANN), Partial Least-Squares (PLS), and Principal Component Analysis (PCA) [16,17]. The exact type of algorithm being used depends on the nature of the problem and the amount of information inserted. In summary, the different types of ML algorithms are presented in Figure 1a [15]. Below, the main categories are described. Supervised ML is a type of ML in which the computer tries to extract conclusions derived from inserted data, based on a set of input–output data examples. For this purpose, there is a labeled dataset used for training where the machine has to forecast the result [18]. Unsupervised ML algorithms rely on unlabeled datasets, meaning that they are based on input data without requiring predefined input–output pairs [18]. Semi-supervised ML type is a combination of the two algorithms that have been described above, using simultaneously labeled and unlabeled data [18,19]. This model serves the modern need of real-world data processing, which are usually unlabeled [20]. Reinforcement ML techniques allow computational devices to enhance the efficacy in given environmental parameters [21]. This condition-based method is grounded on an award and punishment system to maximize award or reduce punishment [20]. Multi-task learning is a subset of ML that tries to give a unique solution in distinctive problems simultaneously, considering the affinity between them [15]. Ensemble learning is also part of ML, where multiple models are generated and combined in order to address a specific computational intelligence problem [15]. Artificial Neural Networks (AΝΝ) are inspired by the human brain. They consist of layers, including an input layer that receives the inserted data, one or more hidden layers that analyze it, and an output layer that presents the conclusion [15]. The basic structural components of ANN are nodes, which lie within the layers and exchange information with each other [22]. Not all networks have the same number of layers; the more layers the more synthesized data can be analyzed [23]. Hidden layers, in particular, give ANN the ability to process more intricate information, making them more competitive [22]. Finally, the instance-based learning approach categorizes the same sets of data in the training group. What differentiates the instance learning method is the repository of all the inserted clues and the fact that the explanation is given by examining their proximate clues [15].

### 3.2. Deep Learning (DL)

According to Sarker IH, Deep Learning (DL) is a subset of the machine learning (ML) family that is built on Artificial Neural Networks (ANN) [24]. Like ANN, DL is also characterized by layers (input, hidden, output), though the key feature of DL is the multiple hidden layers, enabling hierarchical feature extraction [25]. DL models often show better performance on handling large and complex datasets than traditional ML models [19,26]. This is illustrated in Figure 1b [24].

The main DL algorithms include the following:

- Multilayer Perceptron (MLP): A feed-forward Artificial Neural Network, with the three classic layers or with a potential for more than one hidden output, each of them connected with the following layer with a node [24,27,28]. MLP is more suitable for data escalation [24].
- Convolutional Neural Network (CNN or ConvNet): Incorporates convolutional, pooling, and connected layers, ideal for processing 2D audiovisual data [24,28].
- Long Short-Term Memory Recurrent Neural Network (LSTM-RNN): A repeated ANN pattern, most compatible for consecutive data analysis [24,29].

Less common DL algorithms include the following:

- Self-organizing map (SOM) and Autoencoder (AE): Unsupervised learning models which aim in extent minimization of data [30,31].
- Restricted Boltzmann machines (RBM): Reduces data scale and extracts patterns [32].
- Deep Belief Network (DBN): An unsupervised learning model for hierarchical feature extraction [33].
- Generative Adversarial Network (GAN): Generates new data similar to the input dataset [34].

### 3.3. Network-Based Approaches

Network-based approaches study relations between molecules [protein–protein interactions (PPIs), drug–disease associations (DDAs), and drug–target associations (DTAs)], emphasizing on their location affinities to reveal drug repurposing potentials [9]. The theory of these networks is that drugs near to the molecular site of a disease tend to be more suitable therapeutic candidates than drugs lying far away from the molecular target [35]. Mathematical approaches such as random walks are applied in order to predict these network relationships, supporting that the movement between network nodes depends on weight characteristics of the nodes [36]. Νetwork-based methods are further divided into multiview learning frameworks, heterogeneous knowledge graph mining, and multimodal implementations. Multiview learning frameworks combine different kinds of data to analyze proximate multilevel correlations [37,38]. Examples include Laplacian regularized sparse subspace learning (LRSSL) which is based on drug–drug associations, e.g., chemical information, in order to reveal new drug–disease correlations [39] and deepDR which uses an autoencoder technique to fuse data from DDAs, DTAs, and drug to drug-side effect associations of ten networks in total [40,41]. A more recent example is the multiview learning with matrix completion (MLMC) framework which combines drug information of five sources and disease information of two sources [38]. In heterogeneous knowledge graphs (KGs) mining, the knowledge graphs (KGs) reveal real-world data multilevel correlations between drug molecules, aliment paths, and therapeutic targets [42]. Methodologies like NeoDTI represent an application of KGs mining, in which four networks of data were analyzed to discover new DTAs [43]. TxGNN model is also an example of KGs, which classifies known therapeutic molecules to repurpose them for approximately 20,000 illnesses. More specifically, TxGNN examines the relationships within a medical graph to reveal unknown drug–disease associations and predict therapeutic relevance of candidate molecules. Then, it ranks the repurposed drugs according to these predictions, supporting researchers in efficiently prioritizing drugs for further validation [44]. In contrast to multiview learning frameworks, KGs do not need any extra preparation before data analysis, maintaining all the significant details [9]. Finally, multimodal implementations are another type of network-based approach. What differentiates multimodal implementations from the aforementioned two types of network-based approaches is the fact that they can mix data from different categories, such as KGs, resemblance information, etc., to reach conclusions [45]. Thus, conclusions are more complete, safer, and of higher quality [9]. STAMP-DPI model, which stands for structure aware multimodal drug–protein interaction, and AttentionMGT-DTA, which searches drug–target affinity, are instances of multimodal deep learning techniques [46,47].

### 3.4. Signature-Based Approaches

Signature-based approaches shift the focus from target-based drug development methods to transcriptomics and gene expression aiming to reveal new therapeutic fields [6]. To achieve their goal, signature-based approaches search in gene expression records [6], like the Connectivity Map (CMap) [48] and the Library of Integrated Network-Based Cellular Signatures (LINCS) [49]. The advantage of these methods over the sole objective ones is that they are more successful, because they give a more representative and accurate description of the real situation [50]. However, they are susceptible to din and data loss, due to big records [51]. In order to adverse these disadvantages, deep learning methods are combined with the signature-based models, such as the DeepCE [50] and the DeepCondex [52]. The evolution of single-cell RNA sequencing (RNA-seq) techniques has contributed to signature-based approach, not only in the development of new drugs, but also in repositioning of the existing ones [53]. Drug2cell and ASGARD exemplifies the implementation of RNA-seq in drug repurposing [54,55].

### 3.5. Natural Language Processing (NLP)

Text-mining techniques and Large Language Models (LLM) provide a meaningful contribution to drug development, despite being less frequent than network-based and signature-based methods [9]. They extract valuable information from scientific libraries, electronic health records, and clinical trials, supporting researchers to discover probable repurposing opportunities [9]. Named entity recognition (NER), relation extraction (RE), and entity normalization are the names of some text-mining codes [56,57,58,59,60]. Converter models have been a milestone in NLP development [58]. BERT is a text-mining model, based on converters, which specializes in biosciences [61]. ChatGPT platforms represent the LLM team that have been used in biomedical research for data extraction, hypothesis generation, and application of precision medicine [62,63].

### 3.6. Applications of AI in Drug Repurposing

AI has revolutionized medicine, as it is a valuable tool for almost every medical practice, not only for the discovery of new treatment approaches, but also for prognosis prediction and diagnostic purposes. Moreover, it can play a role in monitoring the patient’s vital signs, providing therapeutic instructions even in clinical examination [64,65]. Especially in drug development and drug repurposing, AI tools are very useful. They can be used either in searching resemblance between drugs and targets/proteins, or in producing the treatment hypothesis and in de novo drug development [9]. Finding similarities between molecules is a key step in drug repurposing and many AI techniques and algorithms contribute to this direction [66]. Both the structure and the function of molecules are examined for similarities [67], based on the principle of drug tendency to attach to molecules alike to the already known ligands [68]. Especially in drug repurposing, drugs are usually examined according to their structure to reveal new meaningful connections, whereas in new drug development, attention is focused on novel targets by protein amino acid sequencing analysis [69]. In this context, representation learning methods can be used. These are machine learning techniques, which use unsupervised data to automatically predict features, without human factor [70]. Autoencoders (AE) are well-known examples of representation learning methods and can produce features propitiously [71]. In addition, the Word2Vec model embeds words by representing them as vectors, so similar words can be near in the spatial field [71]. The Mol2Vec model is a subtype of the Word2Vec oriented to the representation of structural characteristics of molecules by vectors [72]. Inferentially, the steps of representation learning procedures are (1) distinct structural analysis of both drug and target, (2) production of representation, usually via vectors, and (3) prediction of attraction outcomes or new interactions between molecules [73]. Useful for drug repositioning’s purposes are also the molecular fingerprint-based methods, which are applied in structural analysis of drug molecules by searching in molecular banks and representing molecules as vectors [9]. The Extended-Connectivity Fingerprints (ECFPs) or circular or Morgan fingerprints belong in this category, and they deal with a more targeted representation of molecular structures [74]. Also, the PubChem fingerprints search for 881 specific characteristics through PubChem chemistry base [75]. Daylight fingerprints interpret structures based on their pathway [76] and RDKit-2D are vectors for drug description [77]. The Explainable Substructure Partition Fingerprint (ESPF) produces vectors by searching for repeated chemical characteristics on ChEMBL base [78]. The target-based methods analyze the protein chains, also by using vectors like the amino acid composition (AAC) vectors, the conjoint triad protein feature vectors, the quasi sequences which examine amino acid characteristics and the multi-scale local descriptor (MLD) model for vector creation [79,80,81]. Deep learning methods can also be used for drug and target similarity analysis with encoders which are based on introduction of crude series. For example, SMILES (Simplified Molecular Input Line Entry System) consists of symbols erected in a row, standing for chemical molecules [82]. There are also Convolutional Neural Network (CNN)-based DL encoders, like the DeepConv-DTI which combines CNNs with ECFPs and crude inputs of protein series to reveal motifs of DTIs [68] and RNN-based DL encoders such as the DeepAffinity method. The last one uses SMILES and CNN to assume drug–target correlations and RNN models to train the crude input data [83]. Another choice is the transformer-based encoders, like MolTrans framework, which are more suitable for lengthy sequences [60,84]. Another useful AI tool for drug repurposing is chemical graph kernels on compound graphs. The leading characters here are chemical and compound graphs, in which molecules are converted into a Graph Neural Network (GNN), whereas atoms are represented by nodes while bonds between atoms are represented by edges in order to analyze DTIs [85]. Network-based and signature-based methods are very useful in the creation of therapeutic cases because they analyze the illness pathway and its main target, as discussed in respective sections. Finally, NLP models expand the necessary knowledge around an issue by extracting valuable information from the biomedical literature [9].

### 3.7. AI in De Novo Drug Development

AI contributes meaningfully to de novo drug development [86]. AI tools can handle large amounts of information and overcome the high cost of new drug discovery in a more efficient way compared to the traditional methods [87,88]. The new drug must serve the initial goal, which is to create a pattern of molecules that meet the primary need while not disrupting human biology [89]. Tools used in generative AI are Recurrent Neural Networks (RNNs), Variational Autoencoders (VAEs), and Generative Adversarial Networks (GANs) [90,91,92]. All these tools are trained to produce target-oriented stuff based on the input data [93,94]. For example, a substance-aiming dopamine receptor II can be produced by a merge of RNN and reinforcement learning model [95]. Moreover, AI can synthesize structural data, like amino acids of a protein, to produce a 3D representation of proteins, contributing to new drug sketch [96].

## 4. Applications of AI-Driven Drug Repurposing in Different Medical Fields

AI’s contribution is obvious in almost every medical field. Applications of AI have been described in numerous different medical disciplines that affect all human organs and systems. The most ordinary uses of AI tools and AI-guided drug redirecting firstly relate with SARS-CoV-2 (21.62%). Oncology (18.17%), neurological conditions, mainly Parkinson’s and Alzheimer’s disease (5.72%), infections, other than coronavirus (4.18%), and rare diseases (2.63%) are following [1].

### 4.1. Oncology

Cancer is a disease that affects more and more people, with 14 million cancer diagnosis in 2012 (8.2 of which led to death) compared to an estimation of over 20 million cases in 2025 [97]. According to WHO, 10 million cancer-related deaths were recorded in 2020. More specifically, during the same year, the most commonly diagnosed type was breast cancer, while the leading cause of cancer-related death was lung cancer. Unfortunately, 400,000 cases of childhood cancer are estimated each year [98]. The above data support the need for efficient antineoplastic treatments. However, traditional methods of drug discovery require time and bear the risk of unsuccessful completeness of clinical trials. Thus, drug repurposing addresses these difficulties and is a more cost-effective solution which can bypass drug safety studies and lead to new indications of already approved substances [99]. AI integration in drug repurposing can improve and accelerate the procedure by analyzing omics data (e.g., genomics, proteomics), molecular pathways, drug–target interactions, and medical charts emerging new cancer treatment options from existing drugs [6]. More specifically, AI algorithms participate in recognition of new DTIs making the classical laboratory methods quicker and more effective [100]. Orthogonal drug–target space deconvolution is an ML method which uses structural information about both sides (drug and target) to forecast a potential connection [101]. Interaction studies may concern different types of enzymes and receptor families, as in the case of fingerprint techniques and Rotation Forest models [102]. However, Crowdsourcing ML and multi-kernel learning models focus on relations that affect kinase inhibitors [103,104]. DL methods, like convolutional neural networks, also play a role in identification of DTIs, distinguishing DTIs into efficacious and ineffective, based on specific characteristics of each molecular set, that they have learned previously [105]. Various types of network-based techniques use graphs that stand for drugs and proteins in order to reveal associations between them [106]. More specifically, DTiGEMS+ relies on graphs in combination with affinity determination methods [107]. Moreover, methods based on graph neural networks (GNN), like DGraphDTA, employ GNN and a DL model to analyze DTIs grounded on molecular structures [108]. A new method is iDrug, which merges DTIs and drug–disease relationships in one model at the same time, helping at matching redirected drugs with new different types of diseases [109]. In addition, AI tools can improve molecular docking procedures. Molecular docking is a method performed via computer simulation software that examines if a molecule (drug) fits into the connection surface of a target (protein) and how strong this connection is, like testing keys in a lock [110]. AI overcomes the limitations of docking, including the absence of some 3D protein structures and familiar binding substances [111,112]. For example, VirtualKinomeProfiler studies the connections of kinases, based on chemical molecular profiles, to find repurposed drugs for kinase inhibition [113]. CATNIP also serves repurposing goals by incorporating ML techniques to study other types of molecular information except for chemical ones. In other words, by leveraging diverse data types, such as genomic, proteomic, and clinical trial data, CATNIP compares drugs’ properties and predicts similarity scores, uncovering drug pairs with the same clinical indications. Thus, it can prioritize potential candidates for repurposing [114]. There are also ML models that shift the focus from predicting whether a drug can bind to a target to whether it actually works in the right biological context, especially in specific cells or tissues, like tumors. One of the first attempts towards this direction was the NCI-DREAM Drug Sensitivity Prediction Challenge, a combination of supervised ML models, which analyzed genomic data of 53 cells of patients suffering from breast cancer [115]. The top-effective method is BEMKL (Bayesian Efficient Multiple Kernel Learning), which combines many omics data types, carrying the potential of multiple drug analysis at the same time [116]. In the following paragraphs, selected examples of repurposed drugs, via AI technologies, within oncology are presented.

Metformin, an antidiabetic drug, seems to be useful in therapeutic approach of breast, pancreatic, and prostate cancers, as indicated by DSPathNet (Drug-specific Signaling Pathway Network), a DL method that integrates multi-omics data, like gene expression and pathway features [117]. Network analysis methods have shown that statins, which are drugs against dyslipidemia, and lovastatin in particular, are beneficial for patients with gastric cancer due to the inhibition of histone deacetylase 2 (HDAC2), overexpression of which characterizes gastric tumors’ cells [118]. In addition, simvastatin could be a therapeutic option in different kinds of cancers, as it activates the tumor suppressor gene *TP53* [119]. Breast, gastrointestinal, and urinary tract cancers are potential types of cancers for which simvastatin could be beneficial, so a number of clinical trials are being conducted [120]. b-SDTNBI (Balanced Substructure–Drug–Target Network-Based Inference) is another network-based algorithm, which has indicated the antitumor effects of disulfiram in patients diagnosed with breast cancer [121]. From its first use in alcohol addiction, disulfiram is now considered a significant factor not only for breast tumors, but also for colon, prostate, and thyroid cancers [122,123,124].

Mebendazole inhibits the polymerization of tubulin in microtubules, and thus faces infections by helminths. However, this property also appears to have a role in the proliferation of cancer cells [125]. Via molecular docking, proton pump inhibitors also seem useful in anticancer treatment. For instance, pantoprazole tames the proliferation of colorectal cancer cells by acting as a kinase inhibitor [126]. As cancer is a state of inflammation, anti-inflammatory factors could be useful in cancer therapies. Celecoxib is a such factor that inhibits cyclooxygenase-2 (COX-2), which is a key factor in cancer inflammation due to prostaglandins increase. Clinical trials have indicated a positive impact of celecoxib on breast, colon, head and neck, and prostate cancers [127]. Some drugs have already gained approval for oncology conditions, like thalidomide for multiple myeloma and raloxifene for breast cancer [6]. However, clinical trials aim to prove the anticancer properties of other known drugs (Table 1). Despite its unquestionable value, AI-driven drug repurposing still has to face some challenges that arise.

AI models have been mainly trained on preclinical data and genome variations without taking into consideration clinical data and overlooking phenotypic responses. Such a drawback may lead to invalid AI predictions. In addition, data is fragmented across various databases, making it hard to integrate information into unified AI models. Furthermore, polypharmacy considerations and tissue-specific effects can complicate the prediction models [128].

### 4.2. Rare Diseases

Rare or orphan diseases are defined as the diseases that affect a small portion of the population. This particularly means no more than 5 per 10,000 individuals in Europe [129]. Most rare diseases have a long-term course with adverse consequences for patients, including death at young age; it has been reported that 30% of rare diseases in pediatric population lead to death at preschool age [130]. Unfortunately, only 420 of the 7000 (~6%) rare diseases have been addressed with an approved treatment, while in ~95% of them, the critical treatment option still pending [131]. Apart from the complicated nature of these diseases, the scarcity of medical data, the absence of clinical experts into this field, the small number of affected individuals, as well as the financial unviability are all reasons that prevent pharmaceutical companies from investing in drug development for rare diseases [1]. Additionally, more than 99% of new molecules being tested for such rare diseases do not proceed into the clinical trials’ setting [132]. Approximately $2.5 billion and up to 15 years are required for the final patient’s access to a new drug [4]. Thus, drug repurposing, especially with the contribution of AI, is a safe, time-, and money-saving option towards the therapeutic approach of rare diseases. The drug repurposing process starts by selecting the desired molecular target and the potential drug. At the next step, preclinical studies are performed to determine the drug’s mechanism of action and its impact on the target. Then the candidate drug enters clinical studies in phase II, as the necessary information of phase I is already available. Finally, approval applications to regulatory authorities are being submitted [8]. AI can accelerate and improve all the above steps, making drug repurposing, especially for underinvested rare diseases, more effective [133].

For example, URSA^HD^ (unveiling RNA sample annotation) is an ML model which analyzes different types of disease data trying to match unmet needs with existing drugs [134]. ML models were also applied in the search of Pitt–Hopkins syndrome (PTHS) therapy by analyzing potassium and sodium channel blocking [135]. Furthermore, a knowledge graph (KG), like GNBR (Global Network of Biomedical Relationships), combined with literature data, examined potential combinations of known drugs with rare diseases. It concluded with encouraging results for Wilms tumor and sarcoidosis with Trifluoperazine (phenothiazine) and Everolimus (an mTOR inhibitor), respectively [136]. MediKanren is also a KG-based AI model that was involved in the discovery of the approved therapeutic effect of celecoxib and levocarnitine for *RHOBTB2* and *TMLHE* mutations, respectively [137].

AI tools can even be useful in the construction of human organoids to better understand the pathophysiology of rare conditions and discover a promising cure. NEUROrg, a simulation program of human brain based on different kinds of ML algorithms, exemplifies this. More specifically, in the case of metachromatic leukodystrophy (MLD) it reveled combinations of existing drugs, mainly lenvatinib (a tyrosine kinase inhibitor) and olaparib (an antitumor factor), with positive effect on MLD [138]. One step further, the anticancer Farnesyltransferase inhibitors (FTI) are under approval for usage in Hutchinson–Gilford progeria syndrome (HGPS) [139,140,141]. This rare (1 in 20 million cases) genetic disorder causes the formation of progerin, a dysfunctional farnesylated protein [142]. Another clinically tested drug is the anti-IL1 factor, canakinumab, repurposed from rheumatoid arthritis to Muckle Well Syndrome (MWS), which is characterized by high levels of IL-1 inflammatory factor [143]. Lotfi Shahreza M. et al. described Heter-LP (Heterogeneous Label Propagation), which is a semi-supervised ML model based on various networks including drugs, illnesses, targets, and interactions between them including all the potential combinations. It integrates all data into a multi-layered network (data modeling), analyzes the space resemblances (projection), and finally, produces new associations (label propagation) [144]. Its application in Adrenocortical carcinoma (ACC) resulted in the identification of Cosyntropin, Spironolactone, and Flavin adenine dinucleotide as potential treatments for this rare condition [145]. All these paradigms strongly support the need for drug repositioning and especially the fundamental contribution of AI in this effort in order to meet the therapeutic challenges of rare diseases.

### 4.3. COVID-19

COVID-19 pandemic highlighted the urgent need to discover quick, safe, and effective therapeutic approaches. The deadly consequences of a pandemic, like COVID-19, require functional treatment options and do not leave much room for the time-consuming discovery of new drugs. Τhe possibility of drug redirecting was a life-saving choice for this arising threat [146], thus COVID-19 pandemic represents a striking example of its beneficial application. As the 1988 Nobel laureate Sir James Black said, “the most fruitful basis for the discovery of a new drug is to start with an old drug” [147]. Recent AI capabilities to analyze big datasets, compare information and reveal useful conclusions about molecular interactions gave an extra plus to drug repurposing, making it a more countable practice in the fight against challenging and adverse diseases. DL methods are used to identify new DTIs. For example, Molecule Transformer–Drug Target Interaction (MT-DTI) model [148] is a combined DL method that consists of CNN and RNN algorithms using a SMILE technique to define interactions between the market accessed antiviral drugs and SARS-CoV-2 components. Many viral proteins such as the 3CLpro, RdRP, helicase, 3′-to-5′ exonuclease, endoRNAse, and 2′-O-ribose methyltransferase were examined as potential targets of the already approved drugs like atazanavir, remdesivir, efavirenz, ritonavir, and dolutegravir in order to reveal new allies against the COVID-19 threat [148]. ChemAI, which is a DL network, searched in big databases like DrugBank to discover potential repurposed drugs for COVID-19, creating a first file of 30,000 potential candidates [149].

Additionally, 81 redirected drugs were recruited by Gysi and colleagues, who used a GNN method to discover efficient molecules against COVID-19 [150]. Knowledge graph-based approaches include the endeavor of BenovelentAI company and Zeng et al. [151,152]. More specifically, BenovelentAI’s KG indicated the primary used anti-rheumatoid arthritis agent, baricitinib, as an antiviral factor against SARS-CoV-2 due to its ability to restrain the virus’ endocytosis through AKK1 (kinase) blocking [151]. Zeng et al. in collaboration with Amazon computing services, which provided a DL (RotatE) model, developed a KG called CoV-KGE [152,153]. This KG, which was created by 24 million PubMed articles and DrugBank data, indicated 41 potential therapeutic options against COVID-19, including but not limited to dexamethasone, toremifene, and melatonin [152]. Gradient-boosting decision tree (GBDT) examined the affinity between the 3CLpro of SARS-CoV-2 and 8565 drugs, revealing 20 effective candidates for repurposing [154]. For the purposes of molecular docking and with the 3CLpro of SARS-CoV-2 as target, a docking program by Ton et al. culled out the 3 million potential substances to only 1000 repurposed candidates [155]. Many researchers tried to approve that combinational therapy was better than single-agent therapeutic approach. In this context, Identif.AI (Identifying Infectious Disease Combination Therapy with Artificial Intelligence), based on network associations, supported that remdesivir plus ritonavir plus lopinavir were more active against COVID-19 than remdesivir monotherapy [156]. Similarly, network-based techniques braced double-drug approaches, such as sirolimus–dactinomycin, mercaptopurine–melatonin, toremifene–emodin and melatonin–toremifene for COVID-19 [157,158,159].

Furthermore, BIOiSIM was an ML-based simulation program for pulmonary hypertension remedies with Angiotensin-Converting Enzyme (ACE) and calcium channel blockers (CCB) aimed at repurposing and new drug discovery for SARS-CoV-2 [160]. An AI method worth mentioning is the signature-based ASGARD tool, which uses scRNA-seq information for repurposing goals in individuals with serious coronavirus infection [55]. Additionally, Delijewski et al. used a supervised ML model to indicate zafirlukast, a drug against obstructive respiratory disorders, as a promising antiviral factor against SARS-CoV-2 [161]. An unusual AI model established on image-based data is the ImageMol which analyzes images of molecules to predict their attributes. It was able to predict 10 3CL inhibitors and 2/3 of the approved anti-SARS-CoV-2 inhibitors [162]. As a result of AI-driven drug repurposing, clinical trials have been conducted to further confirm the efficacy of artificially selected repurposed drugs for COVID-19.

For instance, clinical trials on remdesivir supported its beneficial impact on hospitalized patients with additional oxygen administration, as they recovered 4 days quicker than the control group [163]. Based on this fact, the Food and Drug Administration (FDA) authorized the emergency use of remdesivir, even though it was originally restricted to patients with severe forms of the disease [164]. As well, a clinical trial on dexamethasone highlighted the decrease in death rates mainly in patients with invasive mechanical ventilation need and less in patients with other types of oxygen therapy except for invasive methods [165]. Combinational therapies were also the subject of several clinical trials. Patients at the first stages of COVID-19 infection benefited from the simultaneous use of melatonin and toremifene [159]. Another advantageous drug pair was the baricitinib–remdesivir combo in patients with advanced coronavirus infection [164].

Apart from its contribution to drug repurposing, AI also reinforced the de novo drug development during the COVID-19 pandemic. Controlled Generation of Molecules (CogMol) is a DL model, based on SMILES and VAEs, which produced thousands of new anti-COVID potentials molecules aimed at spike and nonstructural proteins of the virus [166]. ML tools have improved the vaccine development landscape by creating computational programs, based on the Reverse Vaccinology (RV) strategy, in order to predict the most suitable epitopes for a strong immune response. VaxiJen and Vaxign-ML are ML-powered programs which exemplify the RV procedure [167,168]. During the COVID-19 emergency, these techniques were widely applied to recognize the most effective antigens. To cite an instance, these methods ranked the spike protein (S protein) as the number one vaccination target, following by the nonstructural protein, NSP3 [169]. Additionally, RNN-based methods were used for S protein simulation in order to reveal appropriate antigens [170].

To conclude, all the above support that AI methods were meaningful allies in the fight against COVID-19 pandemic, assisting not only in the discovery of existing or novel antiviral drugs by exploiting the infection’s pathophysiology, but also in the prevention of severe infection by designing effective vaccines.

### 4.4. Neurological Disorders

The development of new drugs for neurological disorders remains a challenging issue. According to the FDA data, the approval rate of novel substances did not show important difference during the decade 2008–2018, although the drug industry has invested in research and development of neurological therapies [171]. However, neurodegenerative diseases, like Alzheimer’s (AD) and Parkinson’s (PD) disease have a significantly negative impact on patients’ lives. AD is characterized by progressive dementia and impairment of cognitive functions and it is expected that up to 150 million individuals will be diagnosed with AD by 2050 [172]. PD usually appears in elderly people and includes mobility and non-mobility symptoms as well as cognitive disorders, causing annual financial hardship for patients and their families up to $14.4 billion in the USA [173]. The global prevalence rate of PD is estimated to be 10 million people by 2030 [174]. Unfortunately, most of the clinical trials on new drugs for AD were terminated [175], whereas the symptomatic therapies with tacrine and rivastigmine gained FDA approval in 1993 and 1998, respectively [176,177]. In the nearby 2021 aducanumab, the first treatment that inhibits AD’s progression was released [178]. Quite similarly, disease-modifying therapies remain a rather unexplored field in PD [179]. All of the above in combination with the already discussed advantages of drug repurposing and AI-based models over the traditional drug development methods reinforce the application of AI-driven drug repositioning in neurological drug discovery as an alternative solution. DL methods such as GNNs are considered very helpful towards this direction [105,180,181]. Pan X. et al. described the DL model AI-DrugNet approach, that extracts network-based data by examining drug associations, relationships between proteins, which drugs interact with specific proteins, and which drugs are effective against specific diseases. Using GNNs or CNNs, the model suggests new indications of existing drugs [182]. DeepDR is an example of an AI-DrugNet approach that relies on drug–disease and drug–target network-based data. Through random walk algorithm and VAE, it revealed today’s approved drugs against AD, like risperidone [40].

Similarly, deepDTnet is a more data-driven network-based DL model that examined 15 kinds of biological data [183]. Also, ImagMol, which depends on depictions of molecules, performed better results on AD targets such as G protein-coupled receptors (GPCRs), beta-site amyloid precursor protein cleaving enzyme 1 (BACE1), and kinases [162,184]. Moreover, the application of AI on genetic data leads to useful tools. DRIAD is an ML model, which merged gene expression data and identified the most suitable already approved drugs for AD [185]. NETTAG is a DL model that combined genomics, proteomics, and other kinds of omics data in order to recognize AD-associated genes and proteins. Hence, it supported the therapeutic effect of four known drugs on AD. More specifically, ibuprofen, ceftriaxone, cholecalciferol, and gemfibrozil act preventively in the AD development [186]. Pathophysiological similarities between neurodegenerative disorders and diabetes mellitus shift the attention towards a potential neuroprotective action of antidiabetic drugs [187,188]. In particular, GLP-1 receptor agonists like exenatide and liraglutide seem to have a positive impact on such neurological disorders by modifying the calcium levels of brain cells, the signal transmission paths of kinase A and kinase B, and by acting as anti-inflammatory factors [189]. Data from clinical trials support that exenatide ameliorates the mobility dysfunction in PD patients [190,191]. Except for mobility improvement, liraglutide also showed a promising influence on cognitive impairment in PD individuals [192].

In addition, several drug candidates have shown potential disease-modifying effects for neurological diseases like PD. As an example, deferiprone, an iron chelation factor, demonstrated efficiency in iron decrease in substantia nigra, as well as mobility improvement of PD patients [193]. Clinical trials on the secretolytic agent ambraxol have implied its repurposability for PD patients [194]. Calcium channel inhibitors, like isradipine, seem to be safe as anti-PD factors; however, more evidence is needed to prove their effectiveness [195]. Computational methods based on AI techniques are used to explore suitable for repurposing targets in multiple sclerosis, like Transient Receptor Potential Ankyrin 1 (TRPA1) calcium channel, the blockade of which protects myelin stability [196]. During the drug repurposing process, attention is needed in order not to come to wrong conclusions. The case of riluzole underscores this. Riluzole is indicated for amyotrophic lateral sclerosis (ALS) and has shown promising results for Huntington’s chorea [197]. Nevertheless, it did not pass the efficacy test due to lack of adjustment of the dose for the new examining indication [198].

### 4.5. Diabetes Mellitus

Diabetes mellitus is characterized by elevated levels of blood glucose (hyperglycaemia) and is divided into many types regarding clinical symptoms, etiology, and treatment [199]. The most common type that affects 90% of diabetics is type II [200], which is related to an unproper cell response to insulin, resulting in constantly high glucose levels [201]. Around 500 million cases of diabetes have been described in 2021 among adults and an approximate increase of 100 and 250 million is expected in 2030 and 2045, respectively [202]. The significant global death rate in adults (12.2%), the increased medical outlays ($966 billion) [200,203], the variety of complications [204,205], and the low compliance of individuals due to possible polypharmacy [206] justify that drug repositioning could be a considerable way of effective therapeutic solutions [207].

Many of the aforementioned AI methods can facilitate the drug repurposing process. New associations between drugs, targets, and diseases can be explored by ML methods (support vector machine—SVM, random forest—RF, Naive Bayesian Classification—NBC) and DL methods (CNNs, GNNs, AEs) through data processing, system examination, and molecular portrayal [71,208,209,210]. Models for prediction of new data–target interactions have been developed, like deepDTnet, which has already been mentioned [183]. Moreover, the Coupled Tensor–Matrix Completion (CTMC) is a matrix-factorization model, which integrates drug and target tensioning, leading to effective drug repurposing [211]. Also, MolAICal relies on DL methods to examine new DTIs [212]. As the similarity between drugs may indicate their common impact on the same situation, AI methods also study associations between drugs. For example, the GCNMK model is based on graph neural network and known drug–drug kernels, whereas the NMDADNN model uses a deep neural network and different types of drug data to reveal new drug–drug interactions (DDIs) [213,214]. New relations between drugs and diseases (drug–disease associations, DDAs) have been studied by matrix-factorization models, such as LMFDA and MSBMF models, while SSGC is a semi-supervised model that combines many kinds of data for a holistic approach of DDAs [215,216,217]. Wankhabe N. et al. fused ML (SVM, RF, NBC) and DL (multi-task deep neural network—MTDNN) models to investigate new inhibitors against dipeptidyl peptidase-4 (DPP-4), a significant enzyme in diabetes pathophysiology. This endeavor revealed five repositioned drugs for diabetes with encouraging results [218].

Antidiabetic drugs are also considered as repurposable drugs for other diseases. Besides their neuroprotective action, antidiabetic agents, which were mentioned in the previous paragraph, have been extensively studied as potential therapeutic agents for cancer. More specifically, metformin seems to have a positive impact on many types of cancer, such as breast, gastrointestinal, lung, and endometrial cancers [219,220,221,222,223,224]. Sulfonylurea drugs like glyburide and gliclazide have potential anticancer properties [225,226]. Similarly, GLP-1 receptor agonists and DDP-4 inhibitors show anticancer effect [227,228,229,230]. SGLT-2-positive cancer cells may be affected by SGLT-2 inhibitors, which block glucose cellular entrance [231].

As for the cardiovascular diseases, SGLT-2 inhibitors and GLP-1 receptor agonists are the main antidiabetic drugs with a reported notable impact by ameliorating myocardial infraction and death rates, preventing kidney disease morbidity, and protecting from stoke episodes [232]. Therefore, diabetes mellitus can be involved on both sides of redirecting: it may either serve as the target of repurposed drugs or provide antidiabetic agents as repurposable drugs for other diseases.

### 4.6. Infectious Diseases

Infections have been a major health problem since the earliest days of humanity. Nowadays, new challenges have emerged, including the increasing resistance of pathogens to existing drugs [233], the underinvestment in research and development for tropical diseases [234], and the uncertain progress of infections, as highlighted by the COVID-19 pandemic [235]. Thus, drug repurposing offers a promising approach to overcome all these issues [236].

AI integration can facilitate the process of repurposing and distinguish the most suitable candidates [237]. ML methods contribute to virtual screening by scanning already known drug–target associations to predict new DTIs (predictive modeling) [238]. Moreover, they examine a great source of data to indicate potential connections (data-driven analysis) [239]. Multiple Linear Regression (MLR), Logistic Regression (LR), RF, and SVM are ML models that assist in target identification for infectious diseases [240]. Regarding DL models, they are the optimal choice for image processing. For example, CNNs study images of viral protein structures in order to reveal potential bonds between them and approved drugs [241]. NLP can also help in data management of infectious diseases. As there are plenty of sources of information concerning infections, NLP extracts clues about existing drugs, targets, and diseases, preventing the omission of key details, which could quite easily be observed with the manual manner of data processing [242,243]. Furthermore, AI addresses the challenge of drug resistance by predicting microbial transformation [244]. In addition, AI techniques meet the need of personalized medicine by focusing on each patient’s profile [245].

Acyclovir, an antiviral factor against HSV infections, exemplifies the AI-driven drug repurposing. Through network and similarity analysis, it was repurposed for CMV infections [246]. Similarly, oseltamivir, which is basically used for influenza A and B cases, has been examined against coronaviruses, like MERS-CoV, via artificial molecular docking [247]. Clinical trials on the anti-HIV1 drugs darunavir and raltegravir have proved their positive effect on COVID-19 and herpes virus plus COVID-19, respectively [248,249]. The immunomodulatory factor mycophenolic acid has shown promising results for Zika virus, Dengue virus, and COVID-19 infections [250]. Chloroquine, which is used in malaria, could be beneficial for HIV and influenza infections [251]. Finally, antipsychotic factors such as chlorpromazine may be beneficial for MERS-Cov and SARS-CoV-2 [252,253].

### 4.7. Pediatrics

The conduct of clinical trials in the pediatric population is limited due to some additional difficulties and challenges that emerge compared to adults. These restrictions include the inability to find pediatric patients who meet the inclusion criteria on rare diseases, which are not so uncommon in the pediatric population. In addition, parental consent is not always easily obtainable, especially when the results are questionable and safety issues are raised. There is also the need to determine an MTD (maximum tolerated dose) that was initially tested in adult trials [254]. Moreover, the diverse pathophysiological profile within the large age range of the pediatric population (0–18 years old), the low-profit investment for the pharmaceutical companies, and ethical concerns pose extra obstacles to the research and development of new pediatric drugs [255]. All of the above indicate that drug repurposing could be an alternative solution [256]. In addition, it has been observed that about 10% of pediatric approved drugs are effectively repositioned drugs in oncology or general pediatric conditions [257].

Blatt and Corey have presented pediatric drugs of Harriet Lane handbook, which have been repurposed for other pediatric uses [257]. They highlighted the accidental discovery of repurposed drugs based on clinical observations or on case reports with coexistence of two pathological conditions that can be treated with one drug. For instance, propranolol, which was originally approved for hypertension, had gained indication for the treatment of hemangioma due to its efficacy in both diseases which co-existed in the same patient [258]. Other drugs repurposed in pediatric oncology are the following: the analgesic factor acetaminophen for hepatoblastoma [259], caffeine which was originally used in neonatal apnea for sarcoma [260], the anti-malarial agent chloroquine for sideroblastic anemia [261], doxycycline that was approved for infections and acne in children > 8 years old for periodontitis, idiopathic pulmonary fibrosis, and vascular abnormalities [262], the antidiabetic drug metformin for cancer prevention [263], lansoprazole, which is primarily used in conditions with high gastric acid secretion, for ITP [264], and the anti-seizure factor valproic acid as an anticancer agent [265].

Except for the pediatric oncology, drug repurposing is a promising therapeutic approach for general pediatrics. For instance, alprostadil was first indicated for PDA (Patent Ductus Arteriosus) closure and showed positive effects on asthma [257,266]. Also, the anti-arrhythmic agent amiodarone proved beneficial for parasitic and fungal infections [257,267,268]. The antibiotic erythromycin seems useful in constipation [257,269] as well as the antiseizure factor phenytoin in epidermolysis bullosa [257,270]. AI in drug repurposing for pediatric diseases cannot be overlooked [255]. However, the scarcity of published data in this field indicates the need for more detailed clinical research in pediatric population.

## 5. Discussion

### 5.1. General Overview

Undoubtedly, drug repurposing represents a promising alternative strategy for the therapeutic management of various diseases. Even the most debilitating and intractable ailments show encouraging responses to repurposed treatments. Concerns about effectiveness, target selection or accuracy, and safety issues are some of the drawbacks of repurposing, which can be diminished by the contribution of computational methods [271].

Indeed, the integration of AI in the drug repurposing process marks a transformative moment in pharmaceutical research and development. AI techniques can process an enormous quantity of data and combine different kinds of information to reveal unknown associations between molecules and distinguish potential treatment options [24]. Throughout this study, several AI tools have been described in detail. Machine learning models, deep learning models, network-based and signature-based approaches, as well as natural language processing are AI techniques contributing to every step of drug discovery, encompassing not only the identification of the suitable target but also the reveal of hidden molecular interactions. In addition, AI models have improved drug repurposing procedures in almost every medical field, emphasizing novel therapeutic solutions even for diseases which currently lack treatment.

Oncology, infectious diseases, neurological diseases, and diabetes mellitus are some of the main domains in which AI-driven drug repurposing projects have been applied. Firstly, in rare diseases where the investment in new drugs development is scarce, the choice of repurposed drugs is crucial. Pandemics, like COVID-19, indicated the urgent need for immediate and effective therapeutic solutions. In such cases, the contribution of AI and drug repurposing has proved meaningful for the reveal of both new antiviral properties and vaccines. This approach has also been described in pediatrics with encouraging results. However, such applications remain limited, highlighting the need for more focused research in this field. Τhe strategic advantages of repositioning over conventional drug development approaches in terms of cost, time efficiency, and risk mitigation have been discussed in the Introduction [4,5,6]. The success rates of repurposed drugs are particularly impressive for rare diseases, with reported success ranging between 30 and 75%, whereas the likelihood of a new drug successfully completing clinical trials is under 10% [8].

Another advantage of AI-driven drug repurposing is that it supports the emerging need for precision medicine, considering the variety of disease versions, the genetic profile, and the individual’s reaction to therapy [272]. Thus, it reinforces the practice of giving the right treatment to the right person at the right time. Moreover, the artificially oriented repositioning complies with the rules of sustainability, due to limited industrial procedures, being an environmentally friendly choice [272]. Lastly, the fusion of real-world data in the training phase of AI models leads to a more realistic illustration of human biology and offers solutions of increased efficiency [273].

### 5.2. Challenges of AI-Driven Drug Repurposing

All the above indicate that AI-driven drug repurposing holds great potential, yet significant challenges still hinder its widespread application. First of all, one of the most important principles in AI training is described by the phrase, “garbage in, garbage out”, which means that the quality of input data defines the outcome, and any kind of bias could be associated with poor results [274]. For instance, training datasets are often noisy and biased towards positive finds, while negative data are limited, affecting the accuracy of training process. In addition, predictions of new molecular interactions require an in-depth understanding of disease pathways, which sometimes is missing, leading to a gap in accurate mapping of drug–target interactions [272]. The previous situation is related to the zero-shot drug repurposing problem, where AI models must discover novel drug–disease relationships without prior examples, either due to the absence of effective treatment or due to the irrelevance between the original indication of the examined drug and the new indication [44]. Biological processes are complex and often context-dependent, for example, the same drug may have different effects on different tissues or under different conditions, which makes accurate predictions by AI models difficult. Furthermore, the complexity and heterogeneity of biomedical data demand domain expertise to interpret AI results correctly and require further laboratory validation [272]. Moreover, ethical considerations, especially concerning patient data privacy, arise [6]. The handling of patient data by AI systems raises ethical concerns, particularly when datasets include sensitive genetic or behavioral information. Thus, ensuring compliance with data protection and security laws is crucial [272]. The opaque internal working process of AI models often raises questions about their transparency, making it hard to trust and adopt AI-based decisions. In addition, lack of access to training databases and unclear methodologies diminish reproducibility and further complicates validation [272]. Effective prompting and careful order issuing are also necessary to relieve these limitations. All these points summarize the ethical concerns arising from the use of AI and highlight the need for more careful and precise procedures during data handling. Furthermore, the field is constrained by the absence of a comprehensive regulatory framework [6]. Although the FDA has established mechanisms like the 505 (b) (2), and the European Medicines Agency (EMA) provides guidance under the Article 10 (5) of Directive 2001/83/EC, there is no international harmonized legal framework, as well as unified guidelines about AI-related concerns [275,276]. Patent considerations also represent an important barrier to the broad implementation of drug repurposing [6]. In case the original patent has already expired, generic competition is allowed. Although “method-of-use” (MOU) patent can protect new indications of the repurposed drugs, they are often weaker and harder to enforce [277]. Hence, the off-label use of generic variations in the repurposed drug continues, preventing pharmaceutical companies from investing in clinical trials for new uses of existing drugs [277]. The European Union grants a 10-year market exclusivity for repurposed drugs covering an orphan condition, as in the case of a new chemical entity. This period can be extended by 2 years, if a Pediatric Investigation Plan (PIP) is included. Repurposed drugs without orphan indications, that are submitted according to article 8 (full dossier) of Directive 2001/83/EC, may gain a 10-year data exclusivity; however, this is uncommon. Data exclusivity for novel indications of well-established drugs is granted for 1 year in the European Union (under article 10 (5) of Directive 2001/83/EC) and 3 years in the United States [275,276]. These limited incentives further complicate the commercial viability of drug repurposing. FDA’s stance on software as a medical device (SaMD) underlies the need for standard regulations. This software is intended to be used for medical purposes, such as diagnosis, monitoring or treatment, without extra hardware medical device and compliance with FDA regulations [278].

### 5.3. Future Directions

Addressing these challenges requires a multifaceted approach. Promoting data exchange between institutions, implementing data pre-processing, and adopting strategies to reduce dataset size could minimize data quality issues, such as noise, missing values, or biased datasets, and enhance performance of AI models [279]. In addition, strong data infrastructures, including well-designed platforms for data sharing and collaborations are necessary. Thus, real-world validation would be easier, integrating AI results into routine clinical practice. Equally important is the development of AI systems that generate interpretable outputs, fostering trust and collaboration between AI and biomedical experts. SHAP and LIME are AI methods, termed explainable XAI, contributing towards this direction [280]. Finally, establishing a detailed regulatory framework and offering incentives to pharmaceutical companies (e.g., longer patent exclusivity intervals, arrangements with generic drug companies) could also increase projects of repurposed drugs [6].

All these improvements can be applied in clinical practice, allowing AI models to provide a more reliable output for repurposed treatments, especially concerning rare diseases. By using interpretable AI models, physicians could understand the mechanisms of repurposing predictions, encouraging trust and adoption in daily clinical practice. Meanwhile, the establishment of a comprehensive regulatory framework would enhance the design of clinical trials that are based on AI-generated predictions. As a result, repurposed drugs could be evaluated more quickly, giving patients earlier access to safe and effective therapeutic options.

## 6. Conclusions

AI-driven drug repurposing represents a revolutionary approach in modern drug discovery, offering a rapid and more cost-effective suggestion compared to the traditional drug development methods. By leveraging diverse AI models from machine learning to deep learning and network- or signature-based algorithms to NLP, researchers can uncover new therapeutic potentials of existing drugs across a wide range of human diseases. Yet, the field of AI-driven drug repositioning faces significant challenges, including the need for deep understanding of molecular mechanisms, poor quality of data, ethical and regulatory uncertainties, and obscure AI results. Addressing these issues requires a combination of actions, which first involves data exchange, data preprocessing, and smaller but superior datasets. Another important step is the development of more easily interpretable AI models, termed explainable AI models. Equally crucial is the establishment of a clear regulatory framework. Currently, there is no comprehensive regulatory path for approving AI-repurposed drugs. Last but not least, governments should offer incentives for pharmaceutical companies to invest in repurposing initiatives. As these scientific, technical, and regulative barriers are progressively addressed, AI-driven drug repurposing aims to play a crucial role in the delivery of safe and effective therapeutics to patients worldwide, particularly in underserved diseases.

## Figures and Tables

**Table 1:** Selected clinical trials for repurposed drugs in cancer and related proliferative conditions (Data derived from clinicaltrial.gov-Trials that have been terminated are not included (Accessed on 20 July 2025).

Trial Code	Condition	Repurposed Drugs	Phase	Type	Status
NCT05977738	Glioblastoma Multiforme (adult), Recurrent Glioblastoma	Pitavastatin calcium	Early Phase I	Interventional	Completed
NCT02770378	Glioblastoma	Temozolomide in combination with: Aprepitant/Minocycline/Disulfiram/Celecoxib/ Sertraline/Captopril/Itraconazole/Ritonavir/Auranofin	Phase I, Phase II	Interventional	Completed
NCT04997811	Myelodysplastic Syndromes (MDS)	Sodium Valproate/Bezafibrate/Medroxyprogesterone vs. Danazol	Phase II	Interventional	Recruiting
NCT03378297	Ovarian cancer	Metformin/Acetylsalicylic acid/Olaparib/Letrozole	Early Phase I	Interventional	Completed
NCT02101008	Melanoma	Disulfiram and Chelated zinc	Phase II	Interventional	Completed
NCT01220973	Prostate cancer	Atorvastatin calcium and Celecoxib	Phase II	Interventional	Completed
NCT01101438	Breast cancer	Metformin	Phase III	Interventional	Completed
NCT03109873	Head and Neck cancer	Metformin	Early Phase I	Interventional	Completed
NCT00582660	Colorectal Adenoma, Colorectal Carcinoma	Celecoxib	Phase II	Interventional	Completed
NCT02896907	Pancreatic Adenocarcinoma, Recurrent Pancreatic Carcinoma, Stage III Pancreatic Cancer, Stage IV Pancreatic Cancer, Unresectable Pancreatic Carcinoma	Chemotherapy (Oxaliplatin/Irinotecan Hydrochloride/Leucovorin Calcium/Fluorouracil) in combination with ascorbic acid	Early Phase I	Interventional	Completed
NCT00462280	Precancerous Condition, Stage 0, Stage I, Stage II Melanoma	Lovastatin	Phase II	Interventional	Completed
NCT00094445	Pancreatic Neoplasms, Adenocarcinoma	Curcumin	Phase II	Interventional	Completed
NCT02227316	Symptomatic Uterine Fibroids and Adenomyosis	Ibuprofen vs. Acetaminophen	Phase IV	Interventional	Completed
NCT02913612	Infantile Hemangioma	Timolol	Phase II	Interventional	Completed
NCT00290758	Breast cancer	Genistein	Phase II	Interventional	Completed
NCT01844583	Solid Tumors, Lymphoma	Alisertib in combination with Esomeprazole or Rifampin	Phase I	Interventional	Completed

## Data Availability

No new data were created or analyzed in this study. Data sharing is not applicable to this article.
